# Anemia at pediatric intensive care unit discharge: prevalence and risk markers

**DOI:** 10.1186/s13613-017-0328-8

**Published:** 2017-10-24

**Authors:** Pierre Demaret, Oliver Karam, Marisa Tucci, Jacques Lacroix, Hélène Behal, Alain Duhamel, Frédéric Lebrun, André Mulder, Stéphane Leteurtre

**Affiliations:** 1 Pediatric Intensive Care Unit, Department of Pediatrics, CHC, Liège, Belgium; 2 Université de Lille, EA 2694 - Santé Publique: épidémiologie et qualité des soins, 59000 Lille, France; 3 Pediatric Critical Care Unit, Geneva University Hospital, Geneva, Switzerland; 4 Division of Pediatric Critical Care Medicine, Children’s Hospital of Richmond at VCU, Richmond, VA USA; 5 Division of Pediatric Critical Care Medicine, Department of Pediatrics, Sainte-Justine Hospital, Université de Montréal, Montreal, Canada; 6 Université de Lille, EA 2694 - Santé Publique: épidémiologie et qualité des soins, Unité de Biostatistique, 59000 Lille, France; 7 Pediatric Intensive Care Unit, CHU Lille, 59000 Lille, France

**Keywords:** Child, Anemia, Erythrocyte, Pediatric intensive care unit, Pediatric, Outcome

## Abstract

**Background:**

Anemia is prevalent at pediatric intensive care unit (PICU) admission and incident during PICU stay, but little is known about anemia at PICU discharge . Anemia after critical illness is an important issue because it could impact post-PICU outcome. We aimed to estimate the prevalence of anemia at PICU discharge and to determine its risk markers.

**Methods:**

This is an ancillary study of a prospective observational study on transfusion practices conducted in the PICU of a tertiary care children’s hospital. All children consecutively admitted to the PICU during a 1-year period were considered for inclusion. Data were prospectively collected from medical charts, except for hemoglobin (Hb) levels at PICU and hospital discharge that were collected retrospectively. Anemia was defined by an Hb concentration below the lower limit of the normal range for age.

**Results:**

Among the 679 children retained for analysis, 390 (57.4%) were anemic at PICU discharge. After multivariate adjustment, anemia at PICU admission was the strongest risk marker of anemia at PICU discharge. The strength of this association varied according to age (interaction): The odds ratio (OR) (95% CI) of anemia at PICU discharge was 4.85 (1.67–14.11) for 1–5-month-old infants anemic versus not anemic at PICU admission, and it was 73.13 (13.43, 398.19) for adolescents anemic versus not anemic at PICU admission. Children admitted after a non-cardiac surgery had an increased risk of anemia at PICU discharge [OR 2.30 (1.37, 3.88), *p* = 0.002]. The proportion of anemic children differed between age categories, while the median Hb level did not exhibit significant variations according to age.

**Conclusions:**

Anemia is highly prevalent at PICU discharge and is strongly predicted by anemia at PICU admission. The usual age-based definitions of anemia may not be relevant for critically ill children. The consequences of anemia at PICU discharge are unknown and deserve further scrutiny.

## Background

Anemia, defined as a concentration of hemoglobin (Hb) below the lower limit of the normal range for age (Table 1) [1], is a common issue in critically ill patients: Approximately two-thirds of critically ill adults are anemic at admission to the intensive care unit (ICU) [2, 3] and up to 98% of them have been reported to be anemic by ICU day 8 [4]. Anemia is frequent in pediatric ICU (PICU) as well: In a large North American multicenter study, one-third of the critically ill children with a PICU stay of at least 2 days were anemic at PICU admission and an additional 40% became anemic during their PICU stay [5].

**Table 1:** Hemoglobin thresholds used to diagnose anemia and anemia prevalence at PICU discharge

Age	Hb threshold to diagnose anemia (g/L)	Proportion of patients anemic at PICU discharge in our study^a^
< 1 month	< 130	39/55 (70.9)
1–5 months	< 95	32/105 (30.5)
6–59 months	< 110	125/231 (54.1)
5–11 years	< 115	70/119 (58.8)
12–14 years	< 120	55/81 (67.9)
≥ 15 years		
Female	< 120	39/46 (84.8)
Male	< 130	30/42 (71.4)
Total		390/679 (57.4)

^a^ Number of anemic children/total number of children in the age category (%)

*Hb* hemoglobin, *PICU* pediatric intensive care unit

Given the high prevalence and incidence of anemia at admission and during ICU stay and given that red blood cell (RBC) transfusion guidelines recommend a restrictive strategy for most critically ill patients [6, 7], it makes sense to wonder about anemia at ICU discharge.

There are scarce data, suggesting that about 85% of adults are anemic when leaving the ICU [8, 9] and that this anemia persists for weeks, most of the patients still being anemic at hospital discharge [9]. It even seems that up to half of adults leaving the ICU with a Hb level < 100 g/L are still anemic 6 months after hospital discharge [10].

Data about anemia at PICU discharge are almost nonexistent. In a single-center Canadian study with large exclusion criteria and no cases of cardiac surgery, 94/392 (24%) children were discharged from PICU with anemia [11]. No other pediatric study on this topic has been published so far.

Anemia is far from trivial and is associated with worse outcomes even in non-critically ill patients. For example, anemia is associated with mortality in African children and a recent meta-analysis of nearly 12,000 children showed that the risk of death falls by 24% for each 1 g/dL increase in Hb [12]. Anemia is associated with neurological outcome. For example, prenatal iron deficiency anemia is associated with a worse mental development of the child at 12, 18 and 24 months of age [13], and a lower mean Hb after a hypoxic–ischemic brain injury following cardiac arrest is associated with a higher odd of unfavorable neurological outcome at hospital discharge [14]. Anemia is also associated with a decreased quality of life and with an increase in healthcare resource utilization [15, 16].

These associations between anemia and worse outcomes explain why the question of anemia after critical illness is of high importance. If anemia is frequent at PICU discharge, then it could play an important role in the post-PICU course.

Outcome after discharge from intensive care is a real challenge for PICU physicians. Mortality rates in PICU are very low (2.4%) as recently reported [17]. Cognitive impairments and physical impairments are currently recognized as potential post-intensive care morbidities of importance [18], and anemia may have an impact on both of them. Limited adult data indicate that patients discharged from ICU with anemia may have a reduced health-related quality of life as compared to the normal population and to non-selected ICU survivors [10]. To our knowledge, no pediatric data have been published so far on this issue.

The first step in assessing the role of anemia at PICU discharge is to determine its prevalence, which is currently unknown. We therefore conducted a study aiming to determine the prevalence of anemia at PICU discharge and its risk markers.

## Methods

### Study design, study site and population

This study is a post hoc analysis of a single-center prospective database aiming to describe transfusion practices in PICU [19, 20].

The PICU of Sainte-Justine University Hospital is a multidisciplinary PICU, serving both medical and surgical specialties. All consecutive admissions to PICU, from April 21, 2009, to April 20, 2010, were prospectively screened for recruitment. A priori defined exclusion criteria were: newborn with gestational age less than 40 weeks at the time of PICU admission, age less than 3 days or more than 18 years at PICU admission, pregnancy or admission just after labor. We excluded a posteriori all children with no Hb level at PICU discharge (as defined below).

### Primary outcome

We retrospectively collected the Hb level at discharge from PICU, defined as follows: the Hb level closest to PICU discharge, collected on a complete blood count after PICU admission and not more than 7 days prior to PICU discharge.

Based on the World Health Organization (WHO) criteria for children aged 6 months and older [21] and on the thresholds proposed in a pediatric textbook for infants younger than 6 months of age [22], we defined anemia as a Hb level < 130 g/L for neonates, < 95 g/L for 1–5-month-old infants, < 110 g/L for 6–59-month-old children, < 115 g/L for 5–11-year-old children, < 120 g/L for 12–14-year-old infants, < 120 g/L for female adolescents aged ≥ 15 years and < 130 g/L for male adolescents aged ≥ 15 years (Table 1).

### Data collection

Trained research assistants prospectively collected data daily in a validated case report form. All information was abstracted from medical charts.

Patient characteristics and baseline data collected within 24 h after PICU admission included age, gender, medical past and admission diagnoses. (More than one diagnosis could be attributed to each patient.) A predictive score of mortality, the Pediatric Risk of Mortality (PRISM score, first version) [23], and a descriptive score of severity of multiple organ dysfunction, the Pediatric Logistic Organ Dysfunction (PELOD score, first version) [24], were used to describe severity of illness at PICU admission.

Several data were prospectively collected during the entire PICU stay. Organ dysfunctions and multiple organ dysfunction syndrome (MODS) were defined according to Goldstein et al. [25]. New MODS was diagnosed if a patient with no organ dysfunction or one organ dysfunction at the time of PICU admission developed two or more organ dysfunctions during PICU stay or if he/she died; progressive MODS was diagnosed if a patient who already had MODS at PICU admission developed dysfunction of at least one other organ during PICU stay or if he/she died. Infections were recorded as per medical notes in the patient chart and by following up all cultures done during PICU stay. Severe sepsis and septic shock were defined according to the definitions published by an International Pediatric Sepsis Consensus Conference [25]. Use of extracorporeal support techniques as well as mortality (death in PICU and in hospital) and the length of stay in PICU and in hospital were recorded.

The Hb concentration at hospital discharge was collected retrospectively and was defined as the Hb level closest to hospital discharge, collected after PICU discharge, but not more than 7 days prior to hospital discharge.

Institutional review board approval was obtained for the collection of the initial database, waiving the requirement for written informed consent due to the observational nature of the study.

### Statistical analysis

Quantitative variables were expressed as mean (standard deviation) if the variable was normally distributed (as assessed graphically and by using the Shapiro–Wilk test) and as median (interquartile range) if not. Qualitative variables were expressed as frequencies and percentages.

Association between anemia at PICU discharge and each patient characteristic at PICU admission or during PICU stay was first studied by bivariate analysis using a logistic regression with a random subject effect (general linear mixed model, GLMM) that allowed us to account for the occurrence of repeated measures per subject (no more than two admissions per subject).

In order to identify independent risk markers of anemia at PICU discharge, we performed multivariable GLMM analysis using the following steps: First, the variables with a *p* value less than 0.2 in bivariate analysis were introduced in a multivariable GLMM. The PRISM score was forced into the model, as we wanted to adjust for the severity of disease. Second, a backward selection at level 0.1 was performed to simplify the model by eliminating variables that were useless in the model. Third, considering the subset of variables selected at the previous step, all the possible first-order interactions were tested in bivariate models. The final multivariable GLMM was obtained by considering the variables selected at the second step and the interactions having a significant level less than 0.1.

## Results

**Fig. 1:**
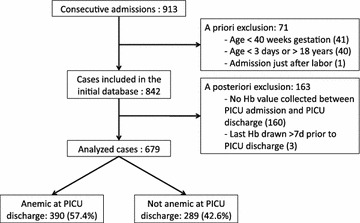
Flowchart of study patients. *PICU* pediatric intensive care unit

Over the 1-year study period, there were 913 consecutive PICU admissions; 71 cases were excluded according to a priori criteria and 163 cases were excluded due to a posteriori criteria (Fig. 1). This left 679 cases for analysis. Children excluded a posteriori differed from those included: They were older (median age (IQR) 53 (12–155) *versus* 39 (6–141) months, *p* = 0.019), were less severely ill (median admission PRISM score 4 (0–6) *versus* 5 (2–9), *p* < 0.001), had less congenital heart disease (8.6% *versus* 23.1%, *p* < 0.001) and had a higher median Hb at PICU admission (122 (108–139) *versus* 110 (95–124) g/L, *p* < 0.001).

### Hemoglobin and anemia at PICU discharge

**Fig. 2:**
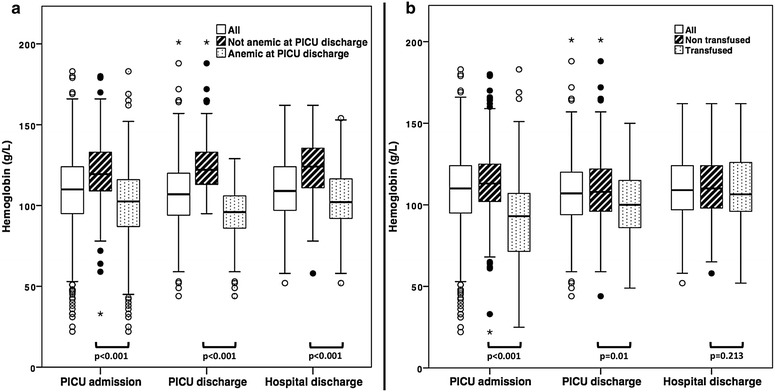
Whisker plots of the hemoglobin level at PICU admission, PICU discharge and hospital discharge for children anemic and non-anemic at PICU admission (**a**) and for children transfused and non-transfused during their PICU stay (**b**). *PICU* pediatric intensive care unit; points and asterisks represent outliers and extreme outliers, respectively

**Fig. 3:**
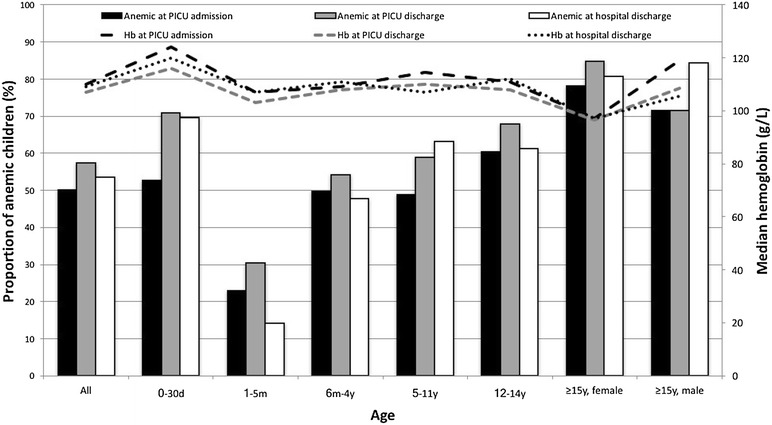
Double *Y*-axis graph on the proportion of anemic children at PICU admission, PICU discharge and hospital discharge according to age, and median Hb level at PICU admission, PICU discharge and hospital discharge according to age. *Hb* hemoglobin, *PICU* pediatric intensive care unit

Among the 679 cases retained for analysis, 390 (57.4%) were anemic at PICU discharge. The last Hb before PICU discharge was collected on the day of PICU discharge for 543 (80%) children, 1 day before PICU discharge for 85 (12.5%) children and 2, 3, 4, 5, 6 and 7 days before PICU discharge for 25 (3.7%), 11 (1.6%), 5 (0.7%), 6 (0.9%), 3 (0.4%) and 1 (0.1%) children, respectively. The median Hb (IQR) at PICU discharge was 96 g/L (86–106) in anemic children and 122 g/L (113–133) in non-anemic children (*p* < 0.001) (Fig. 2a). The proportion of anemic children was higher in neonates (70.9%) and adolescents (male 71.4%, female 84.8%), while it was lower in infants aged 1–5 months (30.5%) (Table 1, Fig. 3).

### Bivariate association between patient, PICU admission or PICU stay characteristics and anemia at PICU discharge

Children anemic at PICU discharge were older than non-anemic children, and they were more likely to suffer from a cancer and less likely to have cyanotic congenital heart disease (Table 2). Children with a higher admission PELOD score showed an increased risk of anemia at PICU discharge as well as those admitted after non-cardiac surgery or due to trauma.

**Table 2:** Bivariate association between patient and PICU stay characteristics and anemia at PICU discharge

	All patients (*n* = 679)	Not anemic (*p* = 289)	Anemic (*n* = 390)	Crude OR (CI_95 %_)	*p* value
*Patient characteristics*					
Age (months)	39 (6–141)	20 (4–89)	57.5 (10.8–159.3)		< 0.001
< 1 month	55 (8.1)	16 (5.4)	39 (10.0)	Reference	
1–5 months	105 (15.5)	73 (25.3)	32 (8.2)	0.18 (0.09, 0.38)	
6–59 months	231 (34.0)	106 (36.7)	125 (32.1)	0.48 (0.25, 0.94)	
5–11 years	119 (17.5)	49 (17.0)	70 (17.9)	0.59 (0.29, 1.20)	
12–14 years	81 (11.9)	26 (9.0)	55 (14.1)	0.86 (0.40, 1.88)	
≥ 15 years	88 (13.0)	19 (6.6)	69 (17.7)	1.47 (0.66, 3.30)	
Gender male	353 (52.0)	147 (50.9)	206 (52.8)	1.08 (0.78, 1.50)	0.62
Cancer (*n*, %)	37 (5.4)	10 (3.5)	27 (6.9)	2.08 (0.99, 4.37)	0.048
Congenital heart disease (*n*, %)					0.018
Cyanotic	84 (12.4)	49 (17.0)	35 (9.0)	0.49 (0.29, 0.80)	
Non-cyanotic	73 (10.8)	29 (10.0)	44 (11.3)	1.04 (0.61, 1.77)	
No congenital heart disease	522 (76.9)	211 (73.0)	311 (79.7)	Reference	
*PICU admission day*					
PRISM score					0.35
> 10	125 (18.4)	53 (18.3)	72 (18.5)	0.84 (0.48, 1.46)	
6–10	194 (28.6)	93 (32.2)	101 (25.9)	0.67 (0.40, 1.11)	
1–5	250 (36.8)	101 (34.9)	149 (38.2)	0.91 (0.56, 1.48)	
0	110 (16.2)	42 (14.6)	68 (17.4)	Reference	
PELOD score					0.034
> 20	27 (4.0)	7 (2.4)	20 (5.1)	2.82 (1.09, 7.29)	
11–20	153 (22.5)	56 (19.4)	97 (24.9)	1.71 (1.1, 2.67)	
1–10	271 (39.9)	113 (39.1)	158 (40.5)	1.39 (0.95, 2.03)	
0	228 (33.6)	113 (39.1)	115 (29.5)	Reference	
Lowest Hb (g/L), mean ± SD	108.9 ± 24.3	120.8 ± 20.1	100.4 ± 23.4	0.96 (0.95, 0.96)	< 0.001
Anemia	341 (50.2)	57 (21.1)	284 (75.1)	11.27 (7.63, 16.65)	< 0.001
Lowest platelets count (× 10^9^/L), mean ± SD	234.5 ± 133.9	250.2 ± 114.6	223.2 ± 145.2	0.98 (0.97, 0.99)	0.021
Thrombocytopenia^a^	194 (28.6)	63 (23.4)	131 (34.7)	1.72 (1.18, 2.5)	0.005
Admission diagnosis					
Respiratory disease	239 (35.2)	127 (43.9)	112 (28.8)	0.51 (0.36, 0.72)	< 0.001
Infection	254 (37.5)	120 (41.5)	134 (34.4)	0.74 (0.53, 1.04)	0.079
Non-cardiac surgery	165 (24.3)	48 (16.7)	117 (30.2)	2.16 (1.44, 3.21)	< 0.001
Cardiac surgery	106 (15.6)	47 (16.3)	59 (15.1)	0.91 (0.59, 1.42)	0.69
Seizures	49 (7.2)	27 (9.3)	22 (5.6)	0.58 (0.31, 1.07)	0.081
Any shock	46 (6.8)	16 (5.6)	30 (7.7)	1.44 (0.74, 2.79)	0.28
Trauma	23 (3.4)	19 (4.9)	4 (1.4)	3.62 (1.17, 11.26)	0.027
*PICU stay*					
Transfusion (*n*, %)					
Any transfusion	176 (25.9)	59 (20.4)	117 (30.0)	1.67 (1.14, 2.45)	0.009
Red blood cells	143 (21.1)	45 (15.6)	98 (25.1)	1.83 (1.21, 2.77)	0.005
Plasma	94 (13.8)	39 (13.5)	55 (14.1)	1.06 (0.66, 1.7)	0.81
Platelets	58 (8.5)	13 (4.5)	45 (11.5)	2.73 (1.4, 5.32)	0.004
Respiratory dysfunction	337 (49.6)	159 (55.0)	178 (45.6)	0.69 (0.5, 0.95)	0.025
Cardiovascular dysfunction	14 (2.1)	4 (1.4)	10 (2.6)	1.91 (0.56, 6.55)	0.30
Hematological dysfunction	115 (16.9)	32 (11.1)	83 (21.3)	2.15 (1.35, 3.42)	0.002
Neurological dysfunction	388 (57.1)	162 (56.1)	226 (57.9)	1.09 (0.79, 1.51)	0.60
Hepatic dysfunction	98 (14.4)	33 (11.4)	65 (16.7)	1.55 (0.97, 2.5)	0.069
Renal dysfunction	24 (3.5)	4 (1.4)	20 (5.1)	3.86 (1.25, 11.95)	0.02
New or progressive MODS	112 (16.5)	43 (14.9)	69 (17.7)	1.22 (0.78, 1.89)	0.38
Death in PICU	25 (3.7)	9 (3.1)	16 (4.1)	1.35 (0.56, 3.24)	0.50
Infection (proven or suspected)	301 (44.3)	137 (47.6)	164 (42.3)	0.81 (0.58, 1.12)	0.20
Severe sepsis/septic shock	63 (9.3)	19 (6.6)	44 (11.3)	1.8 (1.00, 3.26)	0.051
Support techniques (*n*, %)					
Mechanical ventilation	350 (51.5)	147 (50.9)	203 (52.1)	1.05 (0.76, 1.45)	0.75
Duration (day), median (Q1–Q3)^b^	2 (1–5)	2 (1–5)	3 (1–5)		
ECMO/Berlin heart	8 (1.2)	4 (1.4)	4 (1.0)	0.74 (0.17, 3.24)	0.68
Plasmapheresis	5 (0.7)	1 (0.3)	4 (1.0)	–	–
Renal replacement therapy	13 (1.9)	1 (0.3)	12 (3.1)	9.12 (1.11, 75.09)	0.04
PICU length of stay (day), median (Q1–Q3)^c^	3 (2–6)	3 (2–6)	3 (2–6)		0.70
PICU stay > 48 h (*n*, %)	450 (66.3)	185 (64.0)	265 (67.9)	1.2 (0.85, 1.68)	0.30

Values are frequencies and percentage unless otherwise specified

*OR* odds ratio, *PICU* pediatric intensive care unit, *PRISM* pediatric risk of mortality, *PELOD* pediatric logistic organ dysfunction, *Hb* hemoglobin, *SD* standard deviation, *MODS* multiple organ dysfunction syndrome, *ECMO* extracorporeal membrane oxygenation, *CI* confidence interval

^a^Odds ratio calculated for an increase of 10 × 10^9^/L

^b^Calculated for patients mechanically ventilated only

^c^Mann–Whitney U test was used for this variable due to its distribution

Anemia and thrombocytopenia at PICU admission were significantly associated with anemia at PICU discharge (OR (95% CI) 11.27 (7.63, 16.65), *p* < 0.001, and 1.72 (1.18, 2.50), *p* = 0.005, respectively). RBC and platelet transfusions during PICU stay were associated with an increased risk of anemia at PICU discharge (OR 1.83 (1.21, 2.77), *p* = 0.005, and 2.73 (1.4, 5.32), *p* = 0.004, respectively), but plasma transfusions were not (*p* = 0.81).

### Incidence of anemia after PICU admission

Hb at PICU admission was available for 648 children. Among the 307 (49.8%) children not anemic at PICU admission, 94 (30.6%) were anemic at PICU discharge. These 94 children accounted for one quarter (94/390, 24.1%) of the children anemic at PICU discharge.

Hb levels at PICU admission, PICU discharge and hospital discharge were available in 372 children. Among the 139 (37.4%) children not anemic at PICU admission, 38 (27.3%) became anemic during PICU stay and 19 (13.7%) became anemic during the hospital stay after PICU discharge; 38 (27.3%) were still anemic at hospital discharge.

### Multivariable analysis

The adjusted association between anemia at PICU admission and anemia at PICU discharge varied according to age (interaction): The strongest association was for adolescents aged 12–14 years (adjusted OR 33.44 (7.91, 141.33)) and ≥ 15 years (adjusted OR 73.13 (13.43, 398.19)), while the lowest association was for infants aged 1–5 months (adjusted OR 4.85 (1.67, 14.11)) (Table 3). Non-cardiac surgery was an independent risk marker of anemia at PICU discharge (OR 2.30 (1.37, 3.88), *p* = 0.002) as well. Renal dysfunction was retained in the final multivariable model, but its association with anemia at PICU discharge was not statistically significant. Admission PRISM score was forced into the multivariate model, but it was not statistically associated with anemia at PICU discharge (*p* = 0.62). All the other variables included in the multivariate analysis were not retained in the final model after backward selection (cancer; congenital heart disease; thrombocytopenia at PICU admission; admission for respiratory disease, infection, seizure or trauma; RBC or platelet transfusion during PICU stay; respiratory, hematological, hepatic or renal dysfunction; severe sepsis/septic shock; renal replacement therapy).

**Table 3:** Multivariable analysis: risk markers of anemia at PICU discharge

	Adjusted OR (CI_95 %_)	*p* value
Anemia at PICU admission × age		0.056
Anemia at PICU admission^a^		
< 1 month	9.32 (1.99, 43.75)	
1–5 months	4.85 (1.67, 14.11)	
6 months–4 years	6.79 (3.58, 12.91)	
5–11 years	9.13 (3.57, 23.37)	
12–14 years	33.44 (7.91, 141.33)	
≥ 15 years	73.13 (13.43, 398.19)	
Non-cardiac surgery	2.30 (1.37, 3.88)	0.002
Renal dysfunction	3.19 (0.85, 11.92)	0.083

*OR* odds ratio, *PICU* pediatric intensive care unit, *CI* confidence interval

^a^For each age category, children anemic at PICU admission are compared to non-anemic children (reference group)

### Patient outcomes after PICU discharge

Children anemic at PICU discharge had a longer length of hospital stay after PICU discharge than non-anemic children (Table 4). Hospital mortality after PICU discharge did not differ between the two groups (*p* = 0.614). When analyzing the 386 children for whom an Hb level at hospital discharge was available, we found that a high proportion of children with anemia at PICU discharge were still anemic at hospital discharge (178/247, 72%). Surprisingly 21% (29/139) of children who did not have anemia at PICU discharge developed anemia before hospital discharge.

**Table 4:** Outcomes after PICU discharge

Outcome	All patients (*n* = 679)	Not anemic (*n* = 289)	Anemic (*n* = 390)	Univariate OR (CI_95 %_)	*p* value
Hospital length of stay after PICU discharge, median (Q1–Q3)^a^	5 (2–13)	4 (2–9)	6 (3–15)		< 0.001
Death in hospital, *n* (%)^a^	12 (1.8)	6 (2.2)	6 (1.7)	0.75 (0.24, 2.34)	0.614
Hb at hospital discharge, median (Q1–Q3)^b^	109 (97–124)	124 (111–136)	102 (92–117)		< 0.001
Anemia at hospital discharge (*n*, %)^b^	207 (54)	29 (21)	178 (72)	9.79 (5.97, 16.1)	< 0.001

Continuous variables are expressed as median (interquartile range)

*OR* odds ratio, *Hb* hemoglobin, *CI* confidence interval

^a^For children discharged alive from PICU

^b^Available for 386 children

The mean rate of Hb recovery between PICU and hospital discharge was 14.3 ± 40 g/L/week for the entire cohort; it was 18.5 ± 39.7 g/L/week for children anemic at PICU discharge and 6.7 ± 39.5 g/L/week for non-anemic children (*p* < 0.001)

### Hemoglobin, transfusion and anemia at PICU admission, PICU discharge and hospital discharge

Children anemic at PICU discharge already exhibited a lower median Hb at PICU admission as compared with children who were not anemic at PICU discharge, and this difference was still significant at hospital discharge (Fig. 2a). Children transfused with RBCs during their PICU stay had a lower median Hb at PICU admission than non-transfused children, but this difference was reduced (even though still significant) at PICU discharge and was no longer significant at hospital discharge (Fig. 2b).

The proportion of anemic children at PICU admission, PICU discharge and hospital discharge showed the same trend through the age categories: This proportion was highest in female adolescents and lowest in infants aged 1–5 months (Fig. 3). However, such an age-dependant variation is not in phase with the trend of the median Hb level through the age categories: The median Hb level was quite stable from one age category to another and did not significantly decrease in 1–5-month-old infants (Fig. 3).

## Discussion

This observational study shows that anemia is frequent at PICU discharge (57% of children included in our study). We identified two independent risk markers of anemia at PICU discharge: anemia at PICU admission (the strongest risk marker, showing an interaction with age) and admission after a non-cardiac surgery. A high proportion (72%) of children discharged from PICU with anemia were still anemic at hospital discharge. Finally, the proportion of anemic children at PICU admission, PICU discharge and hospital discharge varied according to the age categories, while the median Hb level was quite stable through these age categories (Fig. 3).

### Anemia of PICU patients: from admission to hospital discharge… and beyond?

Anemia is highly prevalent at PICU admission and incident during PICU stay [5]. Our study shows that this anemia of critical illness is not limited to the acute phase of the disease but is still a significant issue at PICU discharge and maybe even subsequently.

We found that anemia at PICU admission was the stronger predictor of anemia at PICU discharge. We also found that the strength of this association varied according to age. Several hypotheses may be raised to explain such an interaction: Causes of anemia may differ from one age category to another; the erythropoietic response may vary depending on age; nutritional and/or therapeutic supports may change from the neonatal period to adolescence and may be associated with a different course of anemia according to age.

Our data on anemia at hospital discharge are limited since the Hb level at hospital discharge was available for only 386 children. Keeping this limitation in mind, it is nevertheless noteworthy that 72% of children anemic at PICU discharge were still anemic when discharged from hospital. We do not have data on the Hb levels after hospital discharge. In a single-center Canadian cohort, 69% (43/62) of children anemic at PICU discharge were still anemic at hospital discharge, but anemia resolved within 4–6 months in 28 patients who were subsequently followed up [11]. Bateman et al. studied 19 adult patients having left the ICU with an Hb < 100 g/L [10]; only 47% (9/19) recovered from their anemia after 6 months of follow-up, with a median time to recovery of 11 weeks. In our study, the mean rate of Hb recovery between PICU and hospital discharge was 14.3 ± 40 g/L/week for the whole cohort; it was 18.5 ± 39.7 g/L/week for children anemic at PICU discharge and 6.7 ± 39.5 g/L/week for non-anemic children (*p* < 0.001). The rate usually encountered in healthy adults is 10 g/L/week; to our knowledge, the rate of recovery in healthy children has not been established yet [10]. We do not know whether children included in our study received RBCs, iron or erythropoiesis-stimulating agents after PICU discharge: Thus, the rate of Hb recovery we observed cannot be properly interpreted, but it is possible that children anemic at PICU discharge exhibit a better erythropoietic reaction than their adult counterparts.

Our finding that 21% of children without anemia at PICU discharge developed anemia during the subsequent hospital stay is also of importance. Are the causes and consequences of this anemia occurring after PICU discharge the same as those of anemia persisting after PICU discharge? What follow-up strategies should be implemented at PICU discharge, if any? Further studies are clearly required to clarify the epidemiology of this “post-PICU” anemia, to estimate the duration of this anemia and to better understand its mechanisms and its potential impacts.

### Is the usual age-based definition of anemia relevant in PICU?

The WHO definition of anemia has been used in many studies. However, this definition has some limitations since it was established more than 40 years ago in a small population sample, without documentation of methodology [29]. Other diagnostic criteria have been proposed but none include pediatric criteria [30].

Studies on the distribution of normal Hb range in a healthy pediatric population are scarce and show quite variable results [31–33]. There is no universal definition of anemia in children. The normal range of Hb depends on several variables including age, race and socioeconomic status. The impact of age is largely explained by the transition from fetal Hb to adult Hb during the first weeks of life. This phenomenon, combined with a downregulation of EPO production related to the increase in blood oxygen content and tissue oxygenation delivery following birth, leads to the so-called physiologic anemia of infancy [34].

However, according to our results (Fig. 3), children admitted to PICU do not exhibit such an age-based fluctuation of Hb level. The usual age-based definitions of anemia are thus questionable in the PICU setting. We were surprised to find that the proportion of anemic children varied through the age categories (drop in 1–5-month-old infants) while the median Hb level was relatively constant. Our results raise the question of the appropriate Hb level to diagnose anemia in critically ill children: Further research is required to address this question.

### Red blood cell transfusions and anemia at PICU and hospital discharge

Children included in our study were transfused according to a restrictive strategy, which recommends transfusion for stable non-cyanotic PICU patients only if their Hb level drops below 70 g/L. In the patients retained for our study, including unstable and cyanotic children, the mean Hb level before the first transfusion was 77.7 ± 22.2 g/L [19]. We do not know to what extent this restrictive RBC transfusion strategy may have impacted the Hb level at PICU discharge. It is plausible that a restrictive strategy increases the risk of anemia at PICU discharge. Indeed, in the Transfusion Requirements in Pediatric Intensive Care Unit study, the overall average lowest Hb concentration from randomization to PICU discharge was 87 ± 4 g/L in the restrictive group and 108 ± 5 g/L in the liberal group (*p* < 0.001), while the Hb levels were similar in these two groups at randomization (80 ± 10 g/L versus 80 ± 9 g/L) [35]. On the other hand, patients transfused with RBCs do not seem to exhibit a greater Hb level than non-transfused patients at (P)ICU discharge. Indeed, it has been shown in a large adult cohort study that the mean Hb difference between transfused and non-transfused patients was high at ICU admission but then decreased over time and becomes nonsignificant [2]. We observed the same trend in our study (Fig. 2b).

All in all, it seems that RBC transfusion during PICU stay is not a reliable tool to distinguish between children who will be anemic at PICU/hospital discharge and those who will not, even when a restrictive transfusion strategy is applied.

### Limitations and strengths

Our study has several limitations. First, Hb levels at PICU and hospital discharge were collected retrospectively, which increases the risk of information bias. Second, the date of occurrence of the variables collected during the PICU stay was not documented, and the Hb we used to characterize anemia at PICU discharge could have been collected within the 7 days prior to discharge. It is thus theoretically possible that a variable considered as a risk marker of anemia at PICU discharge actually occurred after measurement of the discharge Hb. However, we believe that the risk of such protopathic bias is reduced since the discharge Hb was collected the day before or on the day of PICU discharge in 628 out of the 679 included patients (92.5%). Third, the Hb on the day of PICU discharge was not available for all patients, and we considered that a Hb level collected 1 to 7 days before PICU discharge should be a good surrogate of the Hb on the day of PICU discharge. This assertion could not be true and could result in information bias leading to an over- or underestimation of the prevalence of anemia at PICU discharge. However, as stated above, the vast majority of our patients had their last Hb collected the day before or on the day of PICU discharge. The risk of such information bias is thus significantly reduced. Fourth, we excluded 163 cases a posteriori that differed from the included cases; this may have induced a selection bias, which may limit the external validity of our study. Fifth, our study was single center, which also limits its external validity; however, our critical care unit is comparable to most multidisciplinary university-affiliated North American PICUs with regard to case mix and severity of illness. Sixth, our database has been collected in 2009–2010 and some practices may have changed since then, so that our study population may not appropriately reflect children discharged from PICU nowadays.

Our study has also several strengths. This is the first study that evaluates anemia at PICU discharge in a large cohort of critically ill children including neonates and children with congenital heart disease. We enrolled in this study all consecutive PICU admissions over a 1-year period, which resulted in a case mix with a limited risk of selection bias and no influence due to seasonal variation. Finally, all the independent variables were collected prospectively, which is a major asset to minimize information bias.

## Conclusions

Anemia is frequent at PICU discharge and is strongly associated with anemia at PICU admission. While previous studies have focused on anemia at PICU admission and during PICU stay, it seems that the anemia of critically ill child is not limited to the acute phase of the critical illness: There seems to be a continuum of anemia from PICU admission to PICU discharge and hospital discharge.

As it is plausible that anemia at PICU discharge is associated with worse outcomes after PICU stay, efforts should be made to better understand its causes and consequences as well as to implement optimal care and follow-up strategies.

## Abbreviations

CI: confidence interval
EPO: erythropoietin
Hb: hemoglobin
IL: interleukin
IQR: interquartile range
MODS: multiple organ dysfunction syndrome
OR: odds ratio
PELOD: pediatric logistic organ dysfunction
PICU: pediatric intensive care unit
PRISM: pediatric risk of mortality
RBC: red blood cell
WHO: World Health Organization
